# Empfehlungen zur Unterstützung von belasteten, schwerstkranken, sterbenden und trauernden Menschen in der Corona-Pandemie aus palliativmedizinischer Perspektive: Empfehlungen der Deutschen Gesellschaft für Palliativmedizin (DGP), der Deutschen Interdisziplinären Vereinigung für Intensiv- und Notfallmedizin (DIVI), des Bundesverbands Trauerbegleitung (BVT), der Arbeitsgemeinschaft für Psychoonkologie in der Deutschen Krebsgesellschaft, der Deutschen Vereinigung für Soziale Arbeit im Gesundheitswesen (DVSG) und der Deutschen Gesellschaft für Systemische Therapie, Beratung und Familientherapie (DGSF)

**DOI:** 10.1007/s00482-020-00483-9

**Published:** 2020-06-02

**Authors:** Urs Münch, Heidi Müller, Teresa Deffner, Andrea von Schmude, Martina Kern, Susanne Kiepke-Ziemes, Lukas Radbruch

**Affiliations:** 1 Klinik für Allgemein- und Viszeralchirurgie, DRK Kliniken Berlin Westend, Berlin, Deutschland; 2 Trauerzentrum Frankfurt am Main, Frankfurt am Main, Deutschland; 3 Klinik für Anästhesiologie und Intensivmedizin, Kinderklinik Sektion Neonatologie und Pädiatrische Intensivmedizin, Universitätsklinikum Jena, Jena, Deutschland; 4 Netzwerk Hospiz- und Palliativversorgung Bonn/Rhein-Sieg, Bonn, Deutschland; 5 Ansprechstellen im Land NRW zur Palliativversorgung, Hospizarbeit und Angehörigenbegleitung (ALPHA Rheinland), Bonn, Deutschland; 6 Caritasverband für die Region Kempen-Viersen e. V., Viersen, Deutschland; 7 Klinik für Palliativmedizin, Universitätsklinikum Bonn, Venusberg-Campus 1, 53127 Bonn, Deutschland

**Keywords:** Corona-Pandemie, Psychische Belastung, Soziale Isolierung, Palliativversorgung, Virtuelle Kontaktmöglichkeiten, Corona pandemic, Psychological burden, Social isolation, Palliative care, Virtual contact options

## Abstract

Die mit der Corona-Pandemie einhergehenden Einschränkungen und Verbote sorgen für psychische, soziale und spirituelle Belastungen bei Patient*innen mit COVID-19, ihren Zugehörigen und den behandelnden Mitarbeitenden im Gesundheitswesen. Patient*innen mit COVID-19 dürfen nicht von ihren Zugehörigen besucht werden, in vielen Krankenhäusern und Pflegeeinrichtungen gelten generelle Besuchsverbote. Viele Unterstützungsangebote sind verringert oder ganz eingestellt worden. Bei anderen Patient*innen mit sehr kritischen und/oder lebenslimitierenden Erkrankungen werden notwendige Behandlungsmaßnahmen aufgeschoben, weil die Ressourcen im Krankenhaus für an COVID-19 Erkrankte freigehalten werden. Diese Menschen bedürfen jedoch des Gefühls der sozialen Verbundenheit mit ihren Zugehörigen. Für Palliativpatienten sollten Ausnahmen von Besuchsverboten ermöglicht werden. Besuche bei Sterbenden sind mit entsprechenden Schutzmaßnahmen auch auf Isolier- oder Intensivstationen möglich. Für isolierte Patient*innen sollten alternative Möglichkeiten überprüft werden, zum Beispiel via Videotelefonie oder über soziale Medien. Nach dem Versterben sollte den Angehörigen unter ausreichenden Schutzmaßnahmen ein Abschiednehmen ermöglicht oder alternative reale oder virtuelle Wege zum Erinnern und Gedenken angeboten werden. Die Mitarbeitenden in den Behandlungsteams sollten kontinuierlich in der Bewältigung der besonderen Belastungen unterstützt werden. Dazu ist neben klaren Kommunikations- und Entscheidungsstrukturen, Kommunikationsschulungen und psychosozialer Unterstützung vor allem die Bereitstellung der bestmöglichen Rahmenbedingungen für die Arbeit erforderlich.

## Abstract (en)

The corona pandemic has led to a number of restrictions and prohibitions, which in turn place large psychosocial or spiritual burdens on patients with COVID-19, their families and relatives and the treating personnel in the healthcare system. Patients with COVID-19 are not allowed to receive visitors and many hospitals and nursing homes have completely banned visitors. Many support services have been reduced or stopped completely. Necessary treatment interventions for other patients with critical and life-limiting diseases have been delayed or suspended in order to free resources for the expected COVID-19 patients; however, these people need to feel social connectedness with their relatives. Palliative care patients should be exempted from any ban on visitors. Families should be able to visit dying patients even on intensive care units or isolation wards, using adequate protective equipment. Alternative options, such as video telephone calls or via social media should be explored for patients in isolation. Families should also be enabled to say goodbye to the deceased with adequate protective equipment or should be offered alternative real or virtual options for remembrance and commemoration. Health care professionals coping with the exceptional stress should be continuously supported. This requires clear communication and leadership structures, communication training, psychosocial support, but most of all optimal framework conditions for the clinical work.

## Einleitung

Die mit dem SARS-CoV-2-Virus und COVID-19 einhergehenden Einschränkungen, Beschränkungen und Verbote sorgen auf vielen Ebenen für psychische, soziale und spirituelle Belastungen mit Auswirkungen auf die Gesundheit. Das gilt insbesondere für die an dem neuartigen Virus schwer Erkrankten und deren Zugehörige (Familienmitglieder, Partner*innen und Nahestehende). Patient*innen mit COVID-19 werden auf der Isolierstation, Intensivstation oder anderen isolierten Bereichen behandelt und dürfen nicht von ihren Zugehörigen besucht werden. Andere Begleitungs- und Unterstützungsangebote (zum Beispiel durch ehrenamtliche Helfer*innen oder Seelsorger*innen) sind aufgrund der Corona-Pandemie in Folge des Besuchsverbots zumeist verringert oder ganz eingestellt worden. Die Aufgabe der psychosozialen Unterstützung dieser Menschen mit ihrer Not und Angst in dieser existenziellen Krisensituation lastet somit größtenteils und zusätzlich zu allen anderen Aufgaben auf den Schultern der Pflegekräfte und Ärzt*innen vor Ort. Darüber hinaus ist selbst bei sterbenden, mit COVID-19 infizierten Patient*innen für Zugehörige kein Abschiednehmen möglich, da diese Patient*innen in vielen dieser Einrichtungen keinen Besuch erhalten können.

Diese Umstände verstärken die ohnehin schon durch den ungewissen Ausgang der Erkrankung bestehende Belastung für Erkrankte und An- und Zugehörige enorm. Sie beeinträchtigen den Prozess der Abschiednahme und können den Trauerprozess erschweren.

Doch nicht nur die Patient*innen mit COVID-19, sondern auch andere Patient*innen mit sehr kritischen und/oder lebenslimitierenden Erkrankungen leiden unter den Folgen der Pandemie. Sie haben Angst, dass sie die notwendigen Behandlungsmaßnahmen nicht mehr erhalten werden, weil die Ressourcen im Krankenhaus für an COVID-19 Erkrankte freigehalten werden. Sie leiden unter den Folgen der Regulierungen zur physischen Distanzierung, zum Beispiel durch Besuchsverbote in Krankenhäusern und Pflegeeinrichtungen. Zudem fühlen sich in der ambulanten Versorgung sterbende Menschen und ihre Zugehörigen zu Hause alleingelassen [1].

Für alle Patient*innen sowie deren Zugehörige müssen Konzepte entwickelt werden, die eine innovative, flexible und patient*innenzentrierte Begleitung in allen vier Dimensionen der Hospiz- und Palliativversorgung (körperlich, psychisch, sozial, spirituell) abdecken. Die Deutsche Gesellschaft für Palliativmedizin (DGP) hat ihre Empfehlungen ergänzt und Informationen zur Symptombehandlung von Luftnot, Angst, Unruhe und Verwirrtheit bei COVID-19 vorgelegt [2].

Neben der Symptomkontrolle ist aber die psychosoziale und spirituelle Begleitung unter den besonderen Umständen der Pandemie von hoher Bedeutung. Im Folgenden werden Empfehlungen zur psychosozialen und spirituellen Begleitung unter Berücksichtigung der derzeit bestehenden Einschränkungen vorgelegt.

## Psychosoziale und spirituelle Begleitung von kritisch kranken, schwerstkranken und sterbenden Patient*innen (mit oder ohne COVID-19)

Aktuell wird besonderer Wert auf den Schutz von vulnerablen Personengruppen (z. B. Bewohner*innen von Pflegeeinrichtungen, schwerstkranken Patient*innen im Krankenhaus) gelegt. Auch wenn die meisten Regulierungen Ausnahmen bei nicht an COVID-19 erkrankten Palliativpatient*innen vorsehen (zum Beispiel für das Land NRW: https://tinyurl.com/wywr5n6), wird dies nicht immer umgesetzt, sodass die Patient*innen isoliert bleiben. Diese physische Isolation und die damit verbundene Einschränkung oder sogar vollständige Unterbindung sozialer Unterstützung bis ans Lebensende betrifft also sowohl Patient*innen mit schwerem Verlauf von COVID-19 wie auch Palliativpatient*innen ohne COVID-19 und kritisch Kranke auf den Intensivstationen. Für beide Gruppen gelten daher die folgenden Empfehlungen.

Diese Menschen

- bedürfen trotz der Isolation des Gefühls der sozialen Verbundenheit mit ihren Zugehörigen und anderen Personen;
- benötigen eine individuelle und patient*innenzentrierte Beratung zu Therapiewünschen und zur Unterstützung bei der Entscheidungsfindung in Bezug auf mögliche Behandlungsmaßnahmen;
- bedürfen der Unterstützung beim Umgang
  - mit der Isolation (die möglicherweise als traumatisch erlebt wird oder alte Traumata reaktiviert),
  - mit der Möglichkeit, nicht direkt Abschied nehmen zu können,
  - mit der Begrenztheit des eigenen Lebens und der Konfrontation mit der eigenen Sterblichkeit,
  - mit der eigenen Trauer aufgrund der möglichen, fortwährend erlebten Verluste,
  - mit den Sorgen um die ihnen nahestehenden Menschen,
  - mit durch die Situation bedingt möglichen Reaktionen wie Angst, Demoralisierung, Stress und Depressivität,
  - bei spirituellen und existenziellen Nöten;
- bedürfen der Beratung
  - zu ihrer sozialen Situation und Unterstützungsmöglichkeiten,
  - zu Patient*innenverfügung, Vorsorgevollmacht und Testament (falls noch nicht vorhanden oder nicht für die neue Situation ausreichend aktuell).

Diese Themen und damit verbundene Fragen und Ängste können sehr gut im Rahmen der Beratung zur gesundheitlichen Versorgungsplanung nach § 132g SGB V erfolgen.

Ausführliche Empfehlungen zur Kommunikation und zur Therapiezielfindung bei Schwerstkranken, zu den Behandlungsmöglichkeiten bei Angst und Depression und zur Begleitung in der Sterbephase finden sich in der S3-Leitlinie „Palliativmedizin bei Patient*innen mit einer nicht heilbaren Krebserkrankung“ [3].

In Einrichtungen des Gesundheitssystems werden in der Vorbereitung zur Pandemie auch Krisenszenarien diskutiert, wenn die Anzahl der Patient*innen die vorhandenen Behandlungsplätze (Intensivbetten, Beatmungsplätze) übersteigt.

Die DGP erreichen seit Beginn dieser Diskussion Nachfragen von verunsicherten Patient*innen, die befürchten, dass aufgrund ihrer Vorerkrankungen ihre Überlebenswahrscheinlichkeit in einer Triage als nicht ausreichend eingestuft wird und sie deshalb in einem solchen Krisenfall von einer Intensivbehandlung ausgeschlossen werden. Sie haben Angst, dass sie einsam ohne Zuwendung und Unterstützung elendig sterben müssen. Hier ist sicherzustellen und entsprechend zu kommunizieren, dass

- jedes Leben gleichrangig behandelt werden soll unabhängig von Alter, Vorerkrankungen und Herkunft (siehe Empfehlung des Deutschen Ethikrats und klinisch ethische Empfehlungen der DIVI und sechs anderer Fachgesellschaften).
- mit einer guten Palliativversorgung belastende Symptome wie Luftnot, Angst oder Verwirrtheit behandelt werden können, sodass Leid ins Erträgliche gelindert werden kann, auch bis an das Lebensende bei sterbenden Patient*innen,
- sämtliche Möglichkeiten ausgeschöpft werden, trotz der Corona-bedingten Einschränkungen psychosoziale und spirituelle Unterstützung und Hilfen vorzuhalten und einzusetzen.

Wenn aufgrund der Corona-Schutzbestimmungen Besuche nicht erlaubt sind, sollte zunächst geprüft werden, ob nicht für die Palliativpatient*innen Ausnahmen ermöglicht werden können. In den Regulierungen der Landes- und Bundesregierungen werden Palliativpatient*innen von den strengen Regeln zur physischen Distanzierung ausgenommen. In vielen Palliativabteilungen und Pflegeeinrichtungen werden Besuche von nahen Angehörigen bei Sterbenden zugelassen, auch wenn sonst keine Besuche im Krankenhaus erlaubt sind. Bei Patient*innen mit nachgewiesener COVID-19-Erkrankung auf Isolier- oder Intensivstation ist dies grundsätzlich auch möglich, zumindest solange die Anzahl der Fälle noch überschaubar ist und Schutzausrüstung für die Angehörigen zur Verfügung gestellt werden kann. In vielen Einrichtungen wird dies allerdings nicht umgesetzt.

Für isolierte Patient*innen und diejenigen Menschen, die unter Besuchsverboten leiden, sollten alternative Möglichkeiten überprüft werden, damit sie mit ihren An- und Zugehörigen über Telefon, Videotelefonie oder soziale Medien kommunizieren können. Dazu können Smartphones oder Tablets mit entsprechenden Apps genutzt werden. In manchen Krankenhäusern werden dafür Tablets eingesetzt, deren Tastatur angemessen desinfiziert werden kann. Das kann die Belastung durch Isolation reduzieren.

Genauso können neben Psycholog*innen, Sozialarbeiter*innen, Psychoonkolog*innen, Psychotherapeut*innen und Seelsorger*innen auch ambulante Koordinator*innen und ehrenamtliche Mitarbeiter*innen der ambulanten Hospizdienste oder anderer Dienste per Telefon oder Videotelefonie Kontakt aufnehmen und halten. Die Einrichtungen der Hospiz- und Palliativversorgung sollten sich lokal vernetzen, um solche Angebote auszubauen und diese möglichst allen bedürftigen Patient*innen anzubieten.

## Zugehörige

Zugehörige sind ebenfalls besonderen Belastungen ausgesetzt. Auch ohne Corona-Pandemie mussten sie bisher verschiedene Rollen in sich vereinen, waren zugleich Unterstützende der Patient*innen wie auch selbst Betroffene, und mussten die Aufgaben und Rollen übernehmen, die der schwer kranke Mensch nicht mehr ausfüllen konnte [4, 5].

In der aktuellen Situation sind sie zusätzlich belastet durch die Isolierung der Patient*innen, wenn die Besuchsmöglichkeiten fehlen. Eine Umarmung ist nicht möglich, keine Begleitung beim Sterben, oft nicht die Möglichkeit, sich noch einmal das zu sagen, was wichtig ist. Dadurch können die Begleitung der Sterbenden, Abschied und Trauer beeinträchtigt werden.

Ist ein Familienmitglied an COVID-19 erkrankt, kommt bei Zugehörigen möglicherweise noch die Sorge dazu, schuld an der Erkrankung zu sein bzw. die Person selbst angesteckt zu haben. Auch das Erleben von Stigmatisierung im sozialen Umfeld kann sie zusätzlich beeinträchtigen.

Zugehörige bedürfen zu Lebzeiten der/des Erkrankten

- wenn möglich virtueller Kontaktmöglichkeiten zu dem nahestehenden erkrankten Menschen, über (möglichst datenschutzkonforme) Videotelefonie oder Apps für Smartphones, Tablets oder Notebooks. Über Apps wie Skype (www.skype.com), Zoom (www.zoom.us) oder andere kostenlose Angebote können Zugehörige mit den Patient*innen sprechen, sich zum Kaffee verabreden, gemeinsam essen, beten, singen oder Gottesdienst feiern. Sie können Bilder und Lieblingslieder schicken oder Videos miteinander teilen. Auch sedierten Patient*innen kann das Telefon neben das Ohr gelegt werden, damit ihnen Zugehörige etwas erzählen können.
- der Hilfestellungen, wie sie trotz Isolation und Besuchseinschränkungen ihren Lieben nahe sein und sich mit ihnen verbunden fühlen können. Gerade wenn Zugehörige eben genannte virtuellen Möglichkeiten nicht nutzen können, gibt es andere Möglichkeiten, Kontakt zu halten, zum Beispiel Karten/Briefe schreiben, Bilder malen oder den Patient*innen Fotos in Erinnerung an die gemeinsame Zeit senden (https://www.netzwerk-brs.de/angebote-w%C3%A4hrend-der-corona-krise/). Solche Gegenstände können von Patient*innen immer wieder in die Hand genommen, betrachtet und gelesen werden. Selbst wenn Patient*innen aktuell nicht zu lesen vermögen, können Mitarbeiter*innen ihnen z. B. Karten vorlesen.
- wenn notwendig und/oder gewünscht der Unterstützung bei möglichen Sinnfragen wie z. B. „Warum ich, warum wir, warum trifft es sie*ihn?“.
- der Unterstützung bei dem Erleben, sich von der Geschwindigkeit der Ereignisse und der Situation überrollt zu fühlen.
- des Kontakts zum Behandlungsteam, am besten über einen festen Ansprechpartner, der regelmäßig einfühlsam über den Zustand des erkrankten Menschen informiert. Letzteres ist allerdings auf Intensivstationen nicht realisierbar.
- der Krisenintervention.
- der Beratungsmöglichkeiten zu sozialrechtlichen und finanziellen Fragestellungen und Problemen.

In die Begleitung der Zugehörigen können neben Psycholog*innen, Psychoonkolog*innen, Psychotherapeut*innen, Sozialarbeiter*innen und Seelsorger*innen insbesondere ambulant Koordinator*innen oder ehrenamtliche Mitarbeiter*innen der ambulanten Hospizdienste oder anderer Dienste eingebunden werden und per Telefon oder Videotelefonie Informationen und Unterstützung bieten.

## Nach dem Versterben

In allen Einrichtungen ist zu evaluieren, ob die Möglichkeit besteht, von an COVID-19 Verstorbenen in der Pathologie unter Einhaltung aller Schutzmaßnahmen Abschied zu nehmen. Nicht überall wird diese Möglichkeit gewährt. Zugehörige benötigen (wenn gewünscht) Unterstützung dabei,

- wie sie auch ohne Leichnam Abschied nehmen können, z. B. mit einem Foto des Verstorbenen (deshalb bedarf es unbedingt eines Fotos vom verstorbenen Menschen), durch Schreiben eines Briefes an den verstorbenen Menschen, durch Gespräche über unerfüllte Bedürfnisse (in einem geschützten Rahmen) oder über das Beste, was man dem verstorbenen Menschen mit auf den Weg geben möchte [6].
- welche Informationen, Apps, Tipps es aktuell im Umgang mit der Trauer gibt.
- wie sie zu Angeboten der Trauerberatung/-begleitung (im Moment telefonisch oder mittels videogestützter Kommunikation) finden können.
- wie sie mit ihren Kindern so kommunizieren, dass Krankheit und Verlust ohne direkte Abschiedsmöglichkeit auch von diesen möglichst ohne ungünstige Nachwirkungen verarbeitet werden können.
- wie sie diejenigen, die von Verlust am stärksten betroffen sind, sensibel und einfühlsam auch in diesen Zeiten unterstützen können.

(Informationen dazu: www.gute-trauer.de, www.netzwerk-brs.de, https://bv-trauerbegleitung.de/angebote/trauerbegleitende/).

Es wäre hilfreich, wenn sich die Behandlungsteams nach Versterben der Patient*in um die Zugehörigen sorgten. Liegt das Einverständnis der engeren Bezugspersonen/der Klinik für eine Nachsorge vor, dann könnten Mitarbeitende aus dem Team spezifisch damit beauftragt werden, diese Zugehörigen spätestens 2 bis 4 Wochen nach dem Versterben der Patientin/des Patienten zu kontaktieren. Sie könnten ihnen in dem Gespräch z. B. einfühlsam zuhören oder Informationen vermitteln, wo sie Unterstützung für ihre möglichen spezifischen Probleme erhalten können.

Sollten Fachkräfte merken, dass Hinterbliebene darunter leiden, wenn ihnen im Moment Familienmitglieder und Freunde nicht körperlich nahe sein können, oder dass die Teilnahme an der Bestattung nur wenigen Personen vorbehalten ist, dann könnten sie Betroffene dazu anregen, dies später nach dem Abklingen der Corona-Pandemie nachzuholen. Eine persönlich gestaltete Gedenkfeier zu Hause oder an einem für die verstorbene Person bedeutungsvollen Ort mit allen Freunden und Nahestehenden kann dabei eine erste Idee sein. Darüber hinaus sollten sich Trauernde nicht ganz zurückziehen, auch wenn Besuche eingeschränkt oder unmöglich sind. Ihr soziales Umfeld kann Beileidsbekundungen, Anteilnahme und Unterstützung über Telefonanrufe, E‑Mails, SMS-Nachrichten oder andere Kanäle wie WhatsApp oder Signal geben. Regelmäßige Gespräche mit Nahestehenden oder anderen Zugehörigen können mit festen Terminen vereinbart werden, auch wenn diese Kontakte dann jeweils nur kurz sind.

Genauso können regelmäßige Kontakte via Telefon oder soziale Medien mit Mitarbeiter*innen der ambulanten Hospizdienste, der Seelsorge oder anderer Trauerberatungsstellen hilfreich sein.

Manchmal hilft die Erinnerung an gemeinsame Erlebnisse und Erfahrungen. Für manche Menschen sind es Geschichten, für andere Gegenstände zum Anfassen oder bestimmte Lieder, Bilder, Fotos etc. Trauernde können solche Erinnerungen sammeln, auch zusammen mit anderen Zugehörigen. Sie können Gegenstände real an einem Ort sammeln (Erinnerungskiste, Fotobuch) oder auch virtuell (Bilder, Texte und Geschichten; siehe zum Beispiel gedenkseiten.de).

In der Trauer sind Ängste häufige Begleiter [7]. Es gibt inzwischen gute Webseiten, die sich mit Angstgefühlen beschäftigen und erklären, was Trauernden in solchen Situationen helfen kann (zum Beispiel www.angst-panik-hilfe.de/panikanfall-umgang.html).

Mit den Einschränkungen infolge der Corona-Pandemie nimmt das Risiko zu, dass Betroffene in Folge einer Verlusterfahrung eine erhöhte psychische Belastung bis hin zu einer „prolonged grief disorder“ (zu Deutsch: anhaltende Trauerstörung) nach ICD-11 (6B42) erleben [8]. Fachkräfte wie Psycholog*innen, Psychotherapeut*innen, Psychoonkolog*innen, Sozialarbeiter*innen und Seelsorger*innen sollten entsprechend im Kontakt mit Zugehörigen auf Risikofaktoren achten [9]. Trauerspezifische Einrichtungen sollten aus diesem Grunde Adressen von speziell geschulten approbierten Psychotherapeut*innen vorhalten und ggf. weiterverweisen.

## Mitarbeiter*innen von Isolierbereichen, Intensivstationen

Nicht nur die Patient*innen und ihre Zugehörigen unterliegen in der Corona-Pandemie besonderen Belastungen, sondern auch die Mitarbeitenden im Gesundheitswesen, sowohl stationär als auch ambulant. Neben der Belastung durch ihre Arbeit sind sie selbst potenziell betroffen, sorgen sich um die eigene Gesundheit und die ihrer Zugehörigen.

Die Mitarbeiter*innen von Isolierbereichen und Intensivstationen haben besonders hohe Belastungen zu tragen [10], aber gerade für diese gibt es trotz dieser Tatsache keine einheitlichen Empfehlungen, Standards und Qualitätskriterien für die psychosoziale Unterstützung bei Großschadenslagen. Die bestehenden Konzepte des Bundesamts für Bevölkerungsschutz und Katastrophenhilfe zur psychosozialen Notfallversorgung von Einsatzkräften sind nicht für diesen Bereich gedacht und entwickelt worden [11].

Die Pandemie als Großschadensereignis ist für Mitarbeiter*innen in den Notfall‑, Isolier- und Intensivbereichen potenziell traumatisierend [12]. Viele Maßnahmen können frühzeitig und im Vorfeld Anwendung finden, um das Risiko empfundener psychischer Belastung zu reduzieren. Zu diesen Maßnahmen zählen:

- klare Kommunikationsstrukturen vorhalten: Es bedarf eines Informations- und Kommunikationskonzepts, nach welchem in regelmäßigen Abständen (z. B. nach den Vorgaben des betrieblichen Pandemie- oder Krisenplans) insbesondere konkrete Informationen zur aktuellen Lage, Regelungen zu Abläufen, Zuständigkeiten, Maßnahmen zur Bewältigung der Lage und mögliche Szenarien, deren Handhabung und den Implikationen für die Behandlungsteams gegeben werden.
- Für viele kurzfristig für den intensivmedizinischen Bereich geschulte Mitarbeiter*innen mögen in der neuen Umgebung andere Dinge wichtiger sein, dennoch besteht nicht nur, aber gerade bei ihnen ein Risiko fachlicher Überforderung, bzw. eines professionellen Hilflosigkeitserlebens aufgrund fehlender kommunikativer Kompetenzen [13]. Hier ist kurzfristige Schulung in hilfreicher Kommunikation für besonders herausfordernde Situationen notwendig. Diese sollte angepasst an die jeweiligen Bedingungen niedrigschwellig erfolgen und leicht verfügbar sein.
- Mitarbeiter*innen dieser Abteilung brauchen ganz besonders unter Stress und hoher Belastung bestmögliche Rahmenbedingungen bei ihrer Arbeit. Das betrifft vor allem Schutzkleidung, Rückzugsräume, verlässliche Pausen und zuverlässig gute Verpflegung [14]. Materielle Ressourcenengpässe sowie zu wenig personelle Ausstattung können ein Gefühl des Ausgeliefertseins bewirken und die psychische Belastung deutlich erhöhen [15].

In den Phasen intensiver psychischer und physischer Beanspruchung der Mitarbeiter*innen ist vor allem darauf zu achten, dass

- möglichst klare Konzepte vor Ort in Bezug auf Entscheidungskonflikte vorhanden sind, die sich an den klinisch ethischen Handlungsempfehlungen der DIVI orientieren. Das reduziert das Risiko zusätzlicher psychischer Belastungen.
- eine professionelle Betreuung in palliativen Situationen durch die Umsetzung von spezialisierter Palliativversorgung (Empfehlung DGP) erfolgt. Wenn möglich sollte die psychosoziale und spirituelle Patient*innen- und Zugehörigenbetreuung durch diejenigen Fachkräfte vor Ort erfolgen, die dem jeweiligen Team bekannt sind [16].

Mitarbeiter*innen können gerade in dieser Phase auf unterschiedlichen Ebenen belastet und stark beansprucht sein, auch jenseits des Arbeitsplatzes. Das kontinuierliche Tragen der persönlichen Schutzausrüstung stellt einen zusätzlichen Stressor dar. Psychosoziale Unterstützung und Hilfestellung soll an den jeweiligen Bedürfnissen der Mitarbeiter*innen der jeweiligen Einrichtungen orientiert sein und für akut Belastete verlässlich verfügbar sein. Dafür ist Folgendes wichtig:

- Ein pragmatisches, niedrigschwelliges Konzept unter Nutzung bestehender Ressourcen [17]. Um Akzeptanz zu erreichen, ist es unbedingt erforderlich, die Mitarbeiter*innen der Isolier- und Intensivstationen in die Planungen mit einzubeziehen. Andernfalls droht ein gutes Konzept am Bedarf vorbeizugehen oder wird nicht akzeptiert.
- Sensibilisierung und Einbeziehung der Führungskräfte. Diese sind kommunikative Schnittstelle zu allen Mitarbeiter*innen und können mit ihrer Haltung und als Vorbild erheblich dazu beitragen, beruflich bedingte psychische Extrembelastungen als normales, aber wichtiges Thema anzusprechen. Das wirkt sich in der Regel positiv auf die Inanspruchnahme von Unterstützungsangeboten aus.
- Inhaltliche Orientierung an dem, was sich bei psychischer Belastung in potenziell traumatisierenden Situationen als hilfreich erweist (AWMF-Leitlinie „Diagnostik und Behandlung von akuten Folgen psychischer Traumatisierung“):
  Förderung von
  - Sicherheit, Beruhigung und Hoffnung bzw. Perspektive [18]
  - Selbsthilfe: sich selbst und das Team wirksam erleben, z. B. durch kurze Interventionen zur physischen und psychischen Entlastung
  - Förderung von Verbundenheit, z. B. durch gemeinsame Rituale
- Das Erleben potenziell traumatisierender Ereignisse kann als Arbeitsunfall bewertet und deshalb in einem BG-Verfahren behandelt werden. Mitarbeiter*innen soll diese Information zugänglich gemacht werden.

## Mitarbeitende in Einrichtungen der Hospiz- und Palliativversorgung

Auch die Mitarbeiter*innen auf den Palliativstationen, in stationären Hospizen, Altenpflegeeinrichtungen und ambulanten (Pflege‑)Diensten, stationären Palliativdiensten und den ambulanten Hospiz- und Palliativdiensten sind in der Pandemie großen physischen, aber vor allem psychischen und existenziellen Belastungen ausgesetzt.

Teilweise oder vollständige Besuchsverbote, Isolation bei vermuteter oder bestätigter Infektion, Aufnahmestopp in Pflegeeinrichtungen, Auflagen wie ein negativer Corona-Test vor Aufnahme in ein Hospiz sorgen dafür, dass das bisherige Selbstverständnis in der Arbeit und der Arbeitsweise massiv an Grenzen stößt.

Ambulante Hospizdienste haben die ehrenamtlichen Begleitungen in Krankenhäusern, Pflegeeinrichtungen oder zu Hause einstellen müssen und können höchstens telefonisch Kontakt halten. Auch die Angebote der Trauerberatung und -begleitung können bestenfalls telefonisch umgesetzt werden. Besuche bei den Zugehörigen, Familiengespräche oder Gruppenangebote sind ebenso wie die Trauercafés eingestellt worden.

Manche Pflegeheime bestehen auf strikten Isolationsmaßnahmen und verweigern den Teams der spezialisierten ambulanten Palliativversorgung den Zutritt. Im ambulanten Bereich ist der Zugang zu Schutzkleidung, FFP2-Atemmasken und Desinfektionsmitteln eingeschränkt, sodass nicht klar ist, wie lange die Vorräte reichen. Dabei wird gerade den Mitarbeitenden in den ambulanten Diensten besonders viel Angst vor Ansteckung entgegengebracht, da sie von Wohnung zu Wohnung fahren müssen.

Dazu kommt die Anspannung der Palliativpatient*innen und ihrer Zugehörigen, die ja zu einer besonders gefährdeten Gruppe gehören und befürchten müssen, dass sie im Falle einer Rationierung keinen Zugang zur Intensivtherapie finden werden.

Für die Mitarbeitenden in diesen Teams gelten dieselben Empfehlungen wie im vorherigen Abschnitt. Die meisten Teams haben schon in ihrer bisherigen Teamstruktur Rituale entwickelt, die entlastend wirken. Hier ist zu prüfen, ob diese Strukturen in der Vorbereitung auf Krisensituationen noch besser verankert oder verändert werden müssen.

In der Versorgung der Palliativpatient*innen müssen die Kommunikationskanäle in der Pandemie angepasst werden. Sofern umsetzbar ist zu empfehlen:

- Videogestützte Kommunikation mit Laptop, Tablet oder Smartphone
- Telefonische Kommunikation
- Direkte Kommunikation in Schutzkleidung
- Ambulant (sofern möglich): Gespräche am Fenster

Für die ersten beiden Möglichkeiten sind den Mitarbeiter*innen die technischen Mittel zur Verfügung zu stellen. Darüber hinaus muss das Angebot zu allen verfügbaren und geeigneten Möglichkeiten bekannt gemacht werden.

Videogestützte und telefonische Kommunikation ermöglichen auch eine Erreichbarkeit aus dem Homeoffice oder die Sicherstellung eines Notdiensts außerhalb der normalen Werktagarbeitszeiten.

## Einbindung von hospizlich-palliativ qualifizierten Mitarbeiter*innen der psychosozialen und spirituellen Versorgung

Von der Deutschen Interdisziplinären Vereinigung für Intensiv- und Notfallmedizin (DIVI) wird der kurzfristige Aufbau einer klinischen psychosozialen Notfallversorgung im Rahmen der Corona-Pandemie empfohlen und die Integration dieser Versorgung in die Krisenpläne der Pandemie (https://tinyurl.com/tpsjdcw).

Sowohl für die Versorgung der Patient*innen mit COVID-19, die nicht intensivmedizinisch versorgt und beatmet werden, wie auch für Palliativpatient*innen ohne COVID-19 und kritisch kranke Patient*innen auf Intensivstationen bedarf es der Einbeziehung von Expert*innen für die psychologische, soziale und spirituelle Versorgung dieser besonders vulnerablen Personengruppen und ihrer Zugehörigen.

In der Hospiz- und Palliativversorgung qualifizierte Fachkräfte verfügen über Wissen und Kompetenzen im Umgang mit existenziellen Situationen wie Sterben, Tod und Trauer [19–21]. Vor allem bei komplexer und intensiv ausgeprägter Problematik (aber nicht nur dort) ist je nach Schwerpunkt des Unterstützungsbedarfs das Hinzuziehen von Sozialarbeiter*innen, Psycholog*innen, Psychoonkolog*innen, Psychotherapeut*innen, Seelsorger*innen, Hospizkoordinator*innen und Trauerberater*innen/-begleiter*innen angezeigt.

Sie verfügen über Möglichkeiten und Techniken,

- dem Gegenüber würdewahrend mit Präsenz, Mitgefühl und Achtsamkeit zu begegnen,
- Emotionen auszuhalten, aufzufangen und in einen möglichst erträglichen Bereich zu führen,
- Ressourcen zu aktivieren, eigene Kraftquellen zu finden und zugänglich zu machen,
- Menschen zu unterstützen, einen eigenen Umgang mit der Situation zu finden,
- zwischen verschiedenen Gruppen (z. B. verschiedenen Berufsgruppen untereinander bzw. Zugehörigen und Mitarbeiter*innen) zu vermitteln und Verständnis füreinander auch in schwieriger Zeit zu finden,
- Menschen dabei zu unterstützen, sich selbstwirksam zu erleben, aber auch Dinge annehmen zu können, die nicht veränderbar sind,
- andere nicht hospizlich-palliativ qualifizierte und/oder erfahrene Kolleg*innen zu schulen, damit diese ebenfalls in diesen Bereichen eingesetzt werden können.
- interkulturelle Kompetenzen anzuwenden.

Diesen Mitarbeiter*innen sollte ebenso wie allen anderen Mitarbeitenden, die an der Behandlung von Patient*innen mit COVID-19 beteiligt sind, zur Selbstfürsorge und Psychohygiene die Möglichkeit von Supervision bzw. Intervision angeboten werden.

### Infobox Weitere Informationen

**Allgemein**

- Deutsche Gesellschaft für Palliativmedizin mit Informationen zu COVID-19
https://www.dgpalliativmedizin.de/neuigkeiten/empfehlungen-der-dgp.html
- Deutscher Hospiz- und Palliativverband mit Arbeitshilfe zu COVID-19
https://www.dhpv.de/tl_files/public/Aktuelles/News/20200316_Arbeitshilfe_Corona_EF.pdf
- DIVI zur psychosozialen Notfallversorgung bei COVID-19
https://www.divi.de/empfehlungen/publikationen/covid-19/1527-divi-empfehlung-step-up-qualifizierung-pflege-covid19-2/file
- DIVI und sechs weitere Fachgesellschaften: Entscheidungen über die Zuteilung von Ressourcen in der Notfall- und der Intensivmedizin im Kontext der COVID-19-Pandemie: Klinisch ethische Empfehlungen
https://www.divi.de/empfehlungen/publikationen/covid-19/1540-covid-19-ethik-empfehlung-v2/file
- Empfehlungen des Deutschen Ethikrats zu COVID-19
https://www.ethikrat.org/fileadmin/Publikationen/Ad-hoc-Empfehlungen/deutsch/ad-hoc-empfehlung-corona-krise.pdf
- Erweiterte S3-Leitlinie „Palliativmedizin“ 2019
https://www.leitlinienprogramm-onkologie.de/fileadmin/user_upload/Downloads/Leitlinien/Palliativmedizin/Version_2/LL_Palliativmedizin_2.0_Langversion.pdf
- S2k-Leitlinie „Diagnostik und Behandlung von akuten Folgen psychischer Traumatisierung“
https://www.awmf.org/uploads/tx_szleitlinien/051-027l_S2k_Diagnostik_Behandlung_akute_Folgen_psychischer_Traumatisierung_2019-10.pdf
- S3-Leitlinine „Psychoonkologie“
https://www.leitlinienprogramm-onkologie.de/fileadmin/user_upload/Downloads/Leitlinien/Psychoonkologieleitlinie_1.1/LL_PSO_Langversion_1.1.pdf
- AWMF-Leitlinie „Diagnostik und Behandlung von akuten Folgen psychischer Traumatisierung“
https://www.awmf.org/leitlinien/detail/ll/051-027.html
- Empfehlungen zur intensivmedizinischen Therapie von Patienten mit COVID-19
https://link.springer.com/article/10.1007%2Fs00063-020-00674-3
- Zur Versorgung und Betreuung von alten, kranken und sterbenden Menschen aus systemischer Sicht mit und ohne COVID-19
https://www.dgsf.org/ueber-uns/gruppen/fachgruppen/pflegen/mit-und-ohne-corona-die-versorgung-und-betreuung-von-alten-kranken-und-sterbenden-menschen-auf-dem-pruefstand

**Kommunikation**

- VitalTalk: Kommunikationsempfehlungen bei COVID-19
https://www.dgpalliativmedizin.de/images/COVID_ready_communication_German-DEUTSCH_V02.pdf

Hinweise für Seelsorger*innen

- Simon Peng-Keller, Traugott Roser, Thomas Kammerer, Isolde Karle, Kerstin Lammer, Eckhard Frick, Fabian Winiger
https://www.covid-spiritualcare.com/

Hinweise für Psycholog*innen und Psychotherapeut*innen

- https://www.dgpalliativmedizin.de/images/COVID_19_Sektion_Psychologie.pdf

**Umgang mit Verstorbenen**

- Robert Koch-Institut zum Umgang mit Verstorbenen
https://www.rki.de/DE/Content/InfAZ/N/Neuartiges_Coronavirus/Verstorbene.html
